# Clinical laboratory analytes and platelet-associated parameters as surrogate markers of subclinical inflammation in latent tuberculosis infection

**DOI:** 10.3389/fimmu.2025.1662454

**Published:** 2025-09-17

**Authors:** Sivaprakasam T. Selvavinayagam, Adukkadukkam Anusree, Yean K. Yong, Sathish Sankar, Asha Frederick, Manivannan Rajeshkumar, Masilamani S. Kumar, Palani Sampath, Ganga Sankar, Chitrali L. Roy, Sree J. Karishma, Amudhan Murugesan, Pachamuthu Balakrishnan, Sakthivel Govindaraj, Siddappa N. Byrareddy, Vijayakumar Velu, Esaki M. Shankar, Marie Larsson, Meganathan Kannan, Sivadoss Raju

**Affiliations:** ^1^ Directorate of Public Health and Preventive Medicine, Teynampet, Chennai, Tamil Nadu, India; ^2^ Blood and Vascular Biology, Department of Biotechnology, Central University of Tamil Nadu, Thiruvarur, India; ^3^ Laboratory Centre, Xiamen University Malaysia, Sepang, Selangor, Malaysia; ^4^ Department of Microbiology, Saveetha Dental College and Hospital, Saveetha Institute of Medical and Technical Sciences, Saveetha University, Chennai, Tamil Nadu, India; ^5^ State TB Office, Teynampet, Chennai, Tamil Nadu, India; ^6^ State Public Health Laboratory, Directorate of Public Health and Preventive Medicine, Teynampet, Chennai, Tamil Nadu, India; ^7^ Infection and Inflammation, Department of Biotechnology, Central University of Tamil Nadu, Thiruvarur, India; ^8^ Department of Microbiology, Government Theni Medical College and Hospital, Theni, India; ^9^ Department of Research, Meenakshi Academy of Higher Education and Research (MAHER), Chennai, India; ^10^ Department of Pathology and Laboratory Medicine, Emory University School of Medicine, Division of Microbiology and Immunology, Emory National Primate Research Center, Emory Vaccine Center, Atlanta, GA, United States; ^11^ Department of Pharmacology and Experimental Neuroscience, University of Nebraska Medical Center, Omaha, NE, United States; ^12^ Division of Molecular Medicine and Virology, Department of Biomedical and Clinical Sciences, Linköping University, Linköping, Sweden

**Keywords:** latent tuberculosis, biomarkers, erythrocyte sedimentation rate, platelet-large cell ratio, interferon-gamma release assay, ferritin

## Abstract

**Background:**

The global burden of latent tuberculosis infection (LTBI), with one-third of the population, poses a significant challenge in the diagnosis and treatment of TB. Household contacts (HHCs) of active TB-infected individuals are one of the major high-risk groups for whom early screening and timely intervention are highly critical to interrupt TB transmission. The subclinical latent infection transitions into active TB disease due to multiple factors. Laboratory diagnostic markers inherent to interferon-gamma release assay (IGRA) positive and negative HHCs may help predict the risk of LTBI and subsequent reactivation. The study aims to identify biochemical and hematological diagnostic markers associated with HHCs and their IGRA status, and to explore the likelihood of clinical laboratory analytes and platelet-associated parameters for use as surrogate markers of subclinical inflammation in LTBI.

**Methods:**

A cross-sectional study was carried out on the HHCs of active TB-infected individuals and healthy controls to determine the association of biochemical and hematological markers with their IGRA status. Blood samples collected from the participants were tested for different laboratory parameters and analyzed by binary regression analysis to determine their efficacy in predicting the development of LTBI.

**Results:**

Erythrocyte sedimentation rate (ESR), mean platelet volume (MPV), D-dimer, platelet-large cell ratio (P-LCR), and platelet distribution width (PDW) were significantly high among LTBI-positive individuals. Among different markers, significant association with LTBI was observed with ESR, PDW, and P-LCR, with their AUC and p values reported as 0.6950 (p=0.0095**), 0.7333 (p=0.0469*), 0.7150 (p=0.0042**), respectively. Binary regression analysis revealed significantly higher odds of LTBI in individuals with elevated ESR (OR = 3.05), PDW (OR = 4.67), MPV (OR = 3.5), and P-LCR (OR = 7.67).

**Conclusion:**

Our study demonstrated clinical laboratory parameters and platelet indices as useful surrogate markers of subclinical inflammation associated with LTBI.

## Introduction

*Mycobacterium tuberculosis* (MTB) is the leading global health problem and a major infectious cause of morbidity and mortality (1). The WHO Global Tuberculosis Report 2024 estimated a total of 11 million people infected with TB in 2023 and identified an increasing trend in the TB incidence rate and achieving the WHO End TB strategy milestone of a 50% reduction by 2025, far from reach (2). The COVID-19 pandemic during 2019–22 was one of the major contributors to the increase in new cases due to setbacks in case identification and treatment (3). Latent TB infection (LTBI) is characterized by an enduring immune response to MTB antigens, despite the lack of gross clinical, radiological, and microbiological indicators (4). While one-third of the population is infected with MTB, there is an estimated 5–15% risk of reactivation, mostly within the first five years of initial infection (5). Attributing to the global TB burden and a large reservoir of LTBI-infected individuals, the WHO devised policy guidelines and recommendations on the management of LTBI within the framework of the WHO End TB Strategy underscores the need for early detection and timely intervention.

The reactivation of a latent infection to an active TB disease involves a gamut of underlying immunological responses leading to incipient and subclinical TB disease states. Currently, the diagnosis of LTBI relies on the WHO-recommended assays, including the Tuberculin Skin Test (TST) and the Interferon-Gamma Release Assay (IGRA), which suffer from several limitations. This includes a requirement of at least two visits, reduced sensitivity among immunocompromised individuals, and false-positivity among vaccinated individuals with TST, high-cost infrastructure, and uncertainty in the results due to low predictability with IGRA and the discordance between the results of TST and IGRA. The tests for LTBI must therefore be carefully interpreted along with other clinical and radiological evidence. Apart from the lack of a gold standard assay for LTBI detection, there is a lack of understanding of host-based diagnostic biomarkers such as hematological markers, metabolites, and other proteins associated with LTBI and indicators that could predict progression to active TB (6, 7). It is of paramount importance to identify potential diagnostic biomarkers across diverse TB-infected populations (8). This would facilitate the development of improved point-of-care (POC) tests applicable in resource-limited settings (9). Because TB tends to impact the economically weakened sections of society significantly, it is imperative to develop accessible and affordable tests for use in endemic areas with resource-constrained laboratories (10–12).

The decreasing levels of RBCs, WBCs, and platelet count, and elevated erythrocyte sedimentation rate (ESR) and C-reactive protein (CRP) that indicate acute inflammation (13) also acted as inflammatory diagnostic markers in pulmonary TB and correlated with disease severity and prognosis (14, 15). Furthermore, many hematological abnormalities, viz., increased levels of fibrinogen and D-dimer, have been reported to correlate with clinical TB (16) and help in distinguishing it from community-acquired pneumonia (17). TB in a systemic hypercoagulable state involves a homeostatic mechanism activating procoagulant factors (18) with elevated thrombin–antithrombin complexes, D-dimer, and fibrinogen (19). However, to date, studies investigating the biochemical and hematological parameters among close contacts of active TB patients in an endemic setting are scarce. Hence, addressing the knowledge gaps in LTBI biomarker research is key to the development of diagnostic tools with improved accuracy and feasibility for use in resource-limited clinical settings. In this study, the biochemical and hematological profiles of HHCs of active TB disease were evaluated and correlated with their IGRA status. We also explored the likelihood of clinical laboratory analytes and platelet-associated parameters for use as surrogate markers of subclinical inflammation in LTBI.

## Methods

### Study design and participants

A cross-sectional study was conducted between January 2023 and March 2023 at the Directorate of Public Health and Preventive Medicine, State Public Health Laboratory, Chennai, India, on the HHCs of active TB patients (n=111) and healthy controls (n=61). Samples were collected during mid-February 2023. The timeline from the date of detection of the index case to the date of sample collection from the HHCs varied, ranging from 4 days to 391 days, with a mean of 68 days. Inclusion criteria for the study participants were adults aged 18 years or older who were HHCs of microbiologically confirmed pulmonary TB cases and who provided informed consent. Individuals with an active TB diagnosis or treatment, those with a history of TB treatment within the past 12 months, pregnancy, chronic diseases such as HIV, cancer, and chronic kidney disease, and those who have ongoing immunosuppressive treatment were excluded from the study. Healthy controls were above 18 years of age with no history of TB, tested negative for the IGRA test, had no known TB exposure, and had no signs or symptoms suggestive of TB during the screening process. The participants in the healthy control group were staff of the Directorate of Public Health and Preventive Medicine, Chennai, India. The study was reviewed and approved by the Directorate of Public Health and Preventive Medicine Ethical Committee, Chennai, India (DPHM/IEC/2023/102, dated: 8^th^ March 2023). All the participants provided written informed consent to participate in the study and for the data to be published.

### IGRA testing

**Figure 1:**
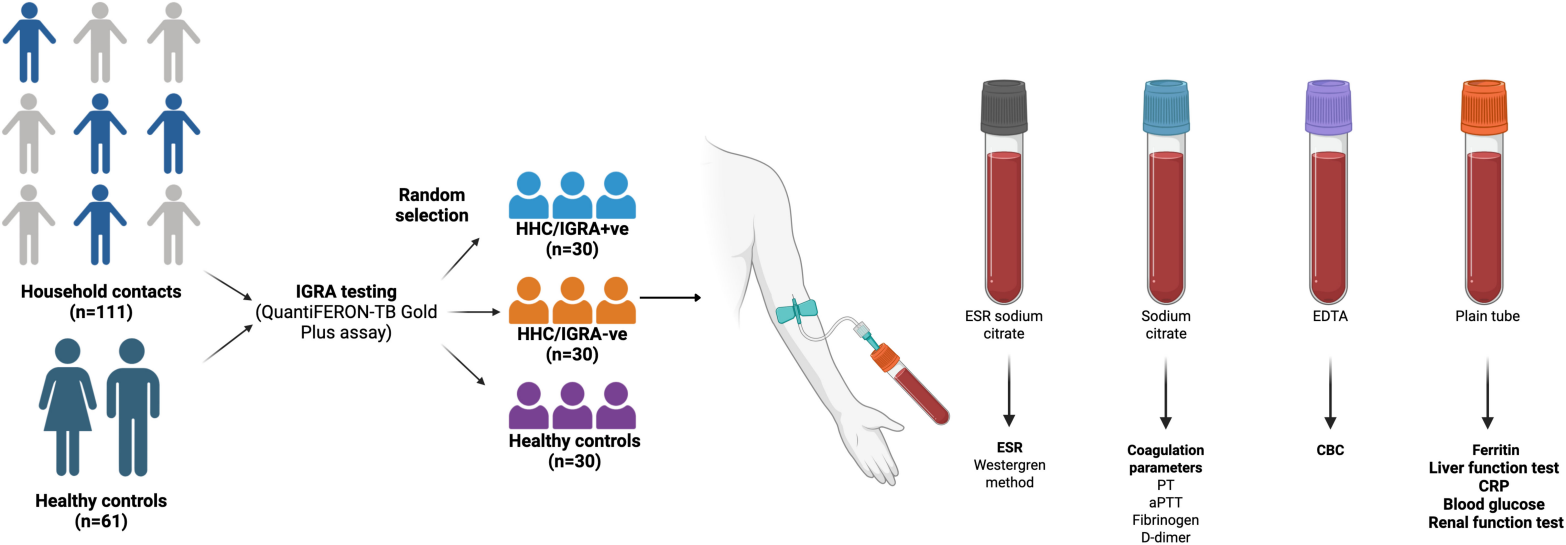
Graphical outline of the study.

All the study participants were tested for LTBI by the IGRA test using QuantiFERON-TB Gold In-Tube Assay (Qiagen GmbH, Hilden, Germany). A total of 60 individuals were randomly selected and categorized into two groups. The first group comprised 30 IGRA-positive (HHC/IGRA+ve), and the second group with 30 individuals who were IGRA-negative (HHC/IGRA-ve). Thirty healthy controls who are not HHC of active TB-infected individuals were enrolled. The groups were not matched for age and sex. The summary of the study is presented in **Figure 1**.

### Sample collection

Ten milliliters of whole blood were drawn from the participants in different vacutainer tubes (BD, Franklin Lakes, USA): 3 mL in plain tube with no additives, 3 mL in EDTA tube, 2 mL in sodium citrate tube, and 2 mL in 3.8% buffered tri-sodium citrate tube, and were centrifuged at 3,000 rpm for 10 minutes. The serum and plasma were made into aliquots in separate tubes for further testing.

### Biochemical and hematological parameters

Sera were processed for testing different biochemical parameters, including blood glucose, ESR, glucose, and CRP. The liver function tests included bilirubin total, aspartate transaminase (AST), alanine transaminase (ALT), alkaline phosphatase (ALP), total protein, albumin, and globulin. The hematological parameters tested include total count (TC), differential counts of neutrophils, lymphocytes, monocytes, eosinophils, and basophils, red blood cell indices including total RBC, hemoglobin, mean corpuscular volume (MCV), mean corpuscular hemoglobin (MCH), mean corpuscular hemoglobin concentration (MCHC), platelet count, red cell distribution width expressed as standard deviation (RDW-SD) and coefficient of variation (RDW-CV), platelet indices including platelet distribution width (PDW), mean platelet volume (MPV), platelet-large cell ratio (P-LCR), procalcitonin (PCT), and neutrophil to lymphocyte ratio (NLR) and coagulation markers, prothrombin time (PT), prothrombin time/international normalized ratio (PT-INR), activated partial thromboplastin time (aPTT), fibrinogen, D-dimer, and ferritin. The renal function test panel consisted of creatinine, urea, blood urea nitrogen (BUN), and uric acid. The biochemical parameters were tested on a fully automated biochemistry analyzer (Siemens Healthcare GmbH, Erlangen, Germany). The hematological profiling was carried out using an automated 5-part Sysmex XN-550 Hematology Analyzer (Sysmex Corporation, Kobe, Japan). The erythrocyte sedimentation rate (ESR) was measured using the classical Westergren method. Serum ferritin was estimated using a fully automated immunoassay system (Siemens Healthcare GmbH, Erlangen, Germany). Coagulation parameters such as prothrombin time (PT), activated partial thromboplastin time (aPTT), fibrinogen, and D-dimer were measured on an Erba Semi-Automatic Coagulation Analyzer (Transasia Bio-Medicals Ltd, Mumbai, India) as per the manufacturer’s instructions.

### Statistical analysis

Comparisons of categorical variables were tested using the Chi-Square test, and continuous variables were tested using a non-parametric Kruskal–Wallis test for multiple group comparisons. If p values were <0.05, 3-way comparisons were subsequently performed separately using Mann–Whitney U tests between the three study groups. The predictive power of diagnostic biomarkers in differentiating HHC/IGRA+ve from healthy controls was examined using receiver operating characteristic (ROC) analysis. The Spearman rank test was used to compare correlations between continuous variables. Biomarkers associated with LTBI were evaluated by binary logistic regression followed by adjusted logistic regression. Statistical analyses were performed using GraphPad PRISM, ver.5.02 (GraphPad Software, San Diego, USA). Binary regression was performed using SPSS, ver.20 (IBM, Armonk, NY). Two-tailed p<0.05 was considered as statistical significance for all tests conducted, and p values <0.05, <0.01, <0.001, and <0.0001 by the Mann-Whitney U test were marked as *, **, ***, and ****, respectively.

## Results

A total of 111 HHCs and 61 healthy controls were recruited for the study. Of 111, 58 (52.3%) were positive for IGRA, 52 (46.8%) were negative, and one sample yielded an indeterminate result. In this study, a randomly chosen HHC/IGRA+ve (n=30), HHC/IGRA-ve (n=30), and healthy controls (n=30) were processed for further analysis. The median (IQR) age of HHC/IGRA+ve was 42 (31 – 54.8), HHC/IGRA-ve was 39.5 (33.5 – 46.3), and healthy controls were 36 (29.5 – 43) in years. All participants were evaluated for biochemical and hematological parameters following confirmation using the IGRA test. The frequency of males in each group was 11 (36.7%), 9 (30%), and 17 (56.7%), respectively. The control group had volunteers who had no HHCs with active TB cases. Further, the frequency of TB exposure duration per day in the HHC/IGRA+ve group was 12 to 18 hrs (56.6%), 6 to 12 hrs (26.6%), and more than 18 hrs (16.6%).

The median BMI (IQR) of the HHC/IGRA+ve, HHC/IGRA-ve, and healthy controls were 26.3 (23.3-29.7), 24.8 (22.1- 28.2), and 25.6 (23.1-28.9), respectively. About 80% of the HHCs reported having BCG vaccination during childhood. The most common comorbid conditions observed were diabetes and hypertension among HHC+ve and HHC-ve individuals, respectively. Among HHC/IGRA+ve individuals, eight (26.6%) individuals presented diabetes, six individuals (20%) in HHC/IGRA-ve, and five (16.7%) in healthy controls, while the rate of hypertension was four (15.4%) in HHC/IGRA+ve, four (13.3%) in HHC/IGRA-ve, and six (20%) among healthy controls. The characteristics of the cohorts are presented in **Table 1**. No significant difference in patient characteristics was observed between the groups. We did not adjust for potential confounders such as diabetes or hypertension, both of which can alter inflammatory and platelet parameters. However, the frequency of these conditions was low and comparable across study groups, reducing, but not eliminating, the risk of bias. Larger studies with higher prevalence of comorbidities are warranted to examine their potential modifying effects.

**Table 1:** Characteristics of the study participants.

Characteristics	HC	HHC/IGRA+ve	HHC/IGRA-ve	p-value
Number	30	30	30	
Age, years	36 (29.5 – 43)	42 (31 – 54.8)	39.5 (33.5 – 46.3)	0.257
Gender, male, n (%)	17 (56.7%)	11 (36.7%)	9 (30%)	0.034
BMI (kg/m^3^)	25.6 (23.1 – 28.9)	26.3 (23.3 – 29.7)	24.8 (22.1 – 28.2)	0.663
BCG vaccination, n (%)	24 (80%)	24 (80%)	23 (76.7%)	0.38
Residential area, Urban, n (%)	15(50%)	15(50%)	15(50%)	0.999

All data reported as median, IQR unless specified. All continuous variables were compared using the Kruskal–Wallis test, while all the categorical variables were compared using the Chi-Square test.

n, number; %, percentage; IQR, interquartile range; HC, healthy control; HHC, household contact; IGRA, interferon-gamma release assay; BMI, body mass index; BCG, Bacille Calmette-Guérin.

### Biochemical and hematological profiling among the study cohorts

**Figure 2:**
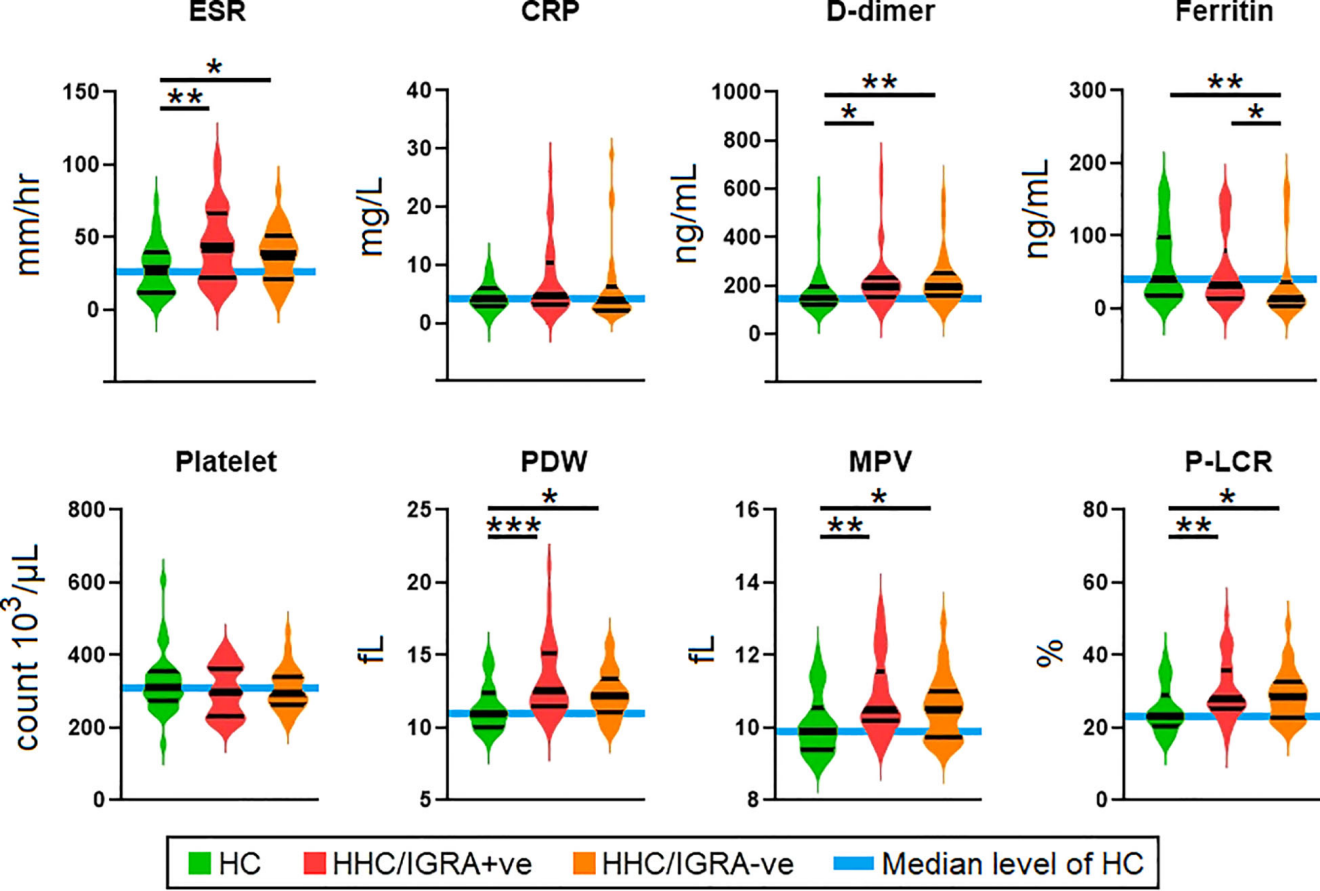
Comparison of the levels of parameters among health controls, HHC/IGRA+ve, and HHC/IGRA-ve individuals. ESR, erythrocyte sedimentation rate; CRP, C-reactive protein; PDW, platelet distribution width; MPV, mean platelet volume; P-LCR, platelet-large cell ratio. Values with *, ** and *** represent p<0.05, <0.01 and <0.001, respectively. Statistically significant deviations between groups are indicated by a horizontal line.

The biochemical and/or hematological parameters showed significant differences between the study groups. **Figure 2** shows the scatter plots for representative biochemical and hematological parameters using the median (interquartile), where a significant difference was observed in ESR, D-dimer, ferritin, PDW, MPV, and P-LCR. Other tested parameters did not show any significant difference across the three groups. ESR, D-dimer, PDW, MPV, and P-LCR were higher in the HHC/IGRA+ve as compared to the healthy controls. The median (IQR) value of all the tested laboratory parameters in the study cohorts is given in **Supplementary Table 1**.

### Efficacy of diagnostic biomarkers in predicting the development of LTBI

**Figure 3:**
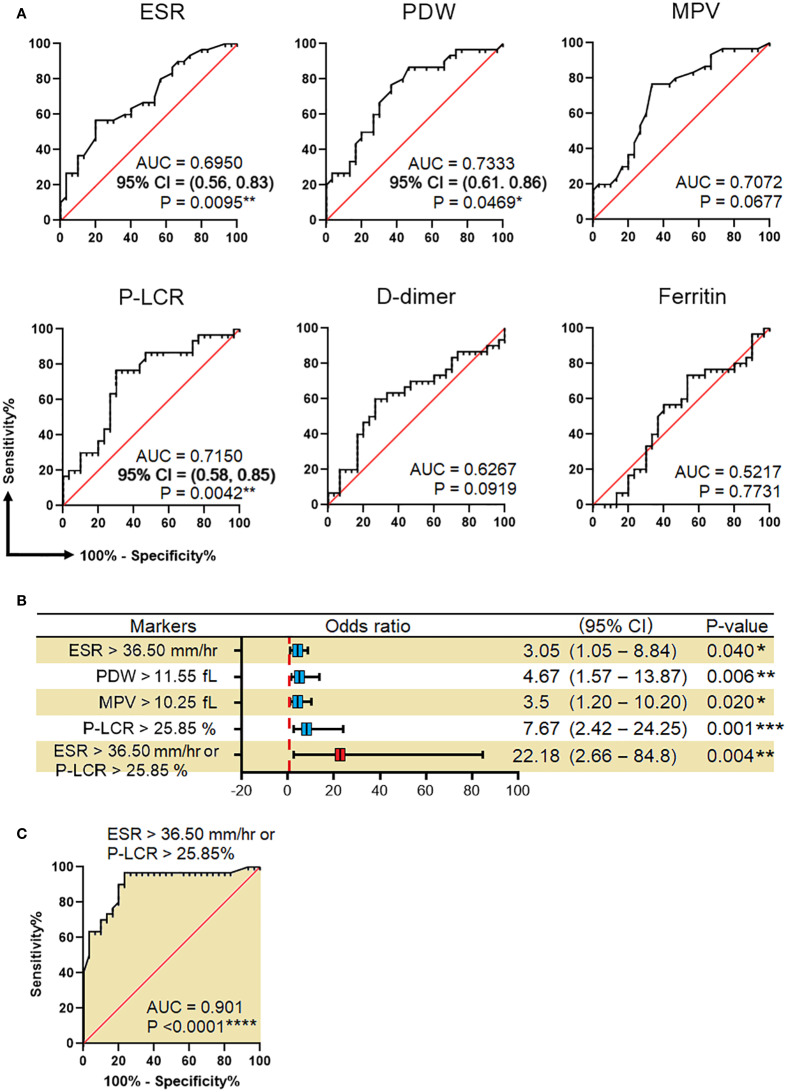
Efficacy of biomarkers in predicting LTBI. **(A)** Receiver operating characteristic (ROC) curves for prediction of LTBI by using ESR, PDW, MPV, P-LCR, D-dimer, and ferritin. **(B)** Association of ESR, PDW, MPV, P-LCR, and either ESR or P-LCR with the risk of LTBI. **(C)** ROC for LTBI prediction by combining ESR >36.50 mm/hr and P-LCR >25.85%. These analyses were done between HC and HHC/IGRA+ve. AUC, the area under the curve; CI, confidence interval; ESR, erythrocyte sedimentation rate; PDW, platelet distribution width; MPV, mean platelet volume; P-LCR, platelet-large cell ratio. Values with *, ** and, *** represent p < 0.05, < 0.01 and < 0.001, respectively.

The predictive power of diagnostic biomarkers in differentiating HHC/IGRA+ve from healthy controls was examined using ROC analysis (**Figure 3**). The sensitivity and specificity of these markers in diagnosing LTBI were compared and reflected as AUC. Of the six markers that showed significance, only three, viz., ESR, PDW, and P-LCR, showed significant association with LTBI, with their AUC and p values reported as 0.6950 (p=0.0095**), 0.7333 (p=0.0469*), 0.7150 (p=0.0042**), respectively. MPV, D-dimer, and ferritin were not significantly associated with LTBI. ROC analysis determined the ESR, PDW, MPV, and P-LCR cut-off as >35.5 mm/hr, >11.55 fL, >10.25 fL, and >25.85%, respectively.

**Figure 4:**
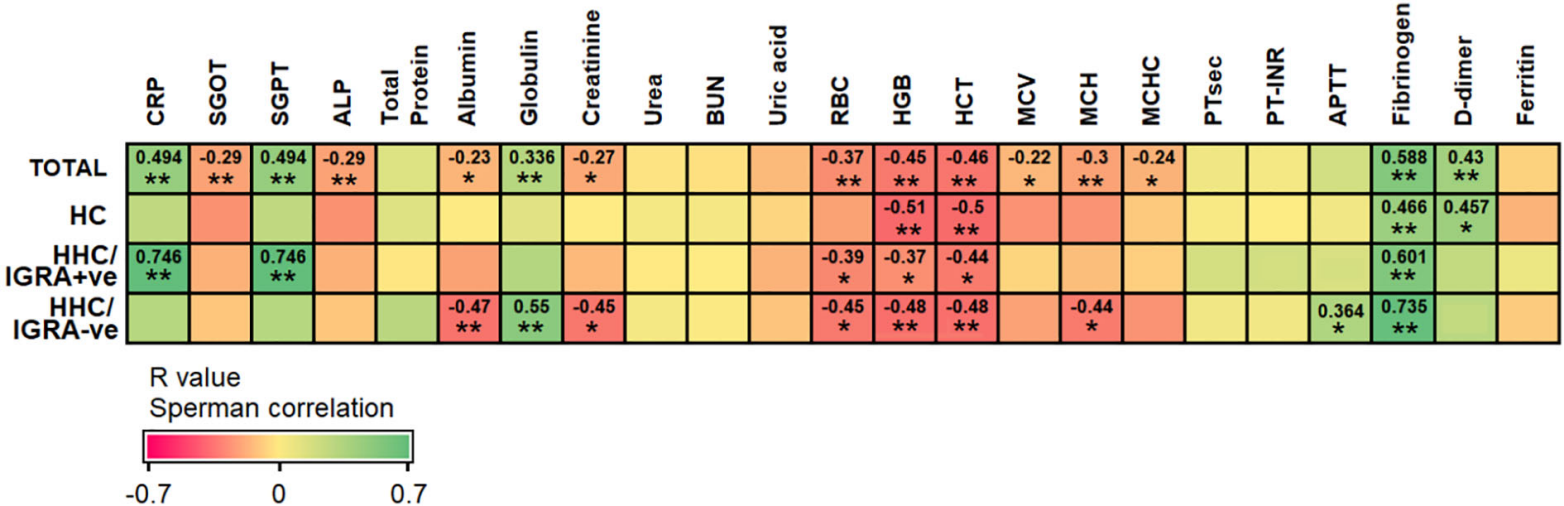
Spearman’s correlation between ESR with other tested parameters among the different groups of participants. The analysis was performed between HCs and HHC/IGRA+ve. Values with *, and ** represent p<0.05, and < 0.01, respectively.

Binary regression analysis showed that the odds of developing LTBI among individuals with ESR, PDW, MPV, and P-LCR greater than their predetermined cut-off were 3.05 (p=0.04*), 4.67 (p=0.006**), 3.5 (p=0.02*), and 7.67 (p=0.001***), respectively. In an attempt to trace the independent diagnostic markers that were associated with LTBI, a multivariate analysis was carried out. Here, the PDW, MPV, and P-LCR were found to be in co-linearity, and hence only ESR and P-LCR were included in the final regression analysis. The study found either ESR or P-LCR to be greater than its pre-determined cut-off and was associated with increased odds of being LTBI+ve by 22-fold with 95% Cl=2.66-84.8, p=0.004**. When combining both ESR >36.5 mm/hr or P-LCR >25.85%, the overall efficacy (measured as AUC) of diagnosing LTBI was 0.906, p<0.0001****. The factors associated with IGRA-ve are mentioned in **Supplementary Table 2**. The Spearman rank test was used to compare correlations between continuous variables. Spearman’s correlation analysis revealed significant correlations between ESR with CRP (r=0.746, p<0.01), ALT (r=0.746, p<0.01), RBC (r=-0.39, p<0.05), hemoglobin (r=-0.37, p<0.05), HCT (r=-0.44, p<0.05) and fibrinogen (r=0.601, p<0.01) in HHC/IGRA +ve individuals (**Figure 4**).

### Association of ferritin and MCHC

**Figure 5:**
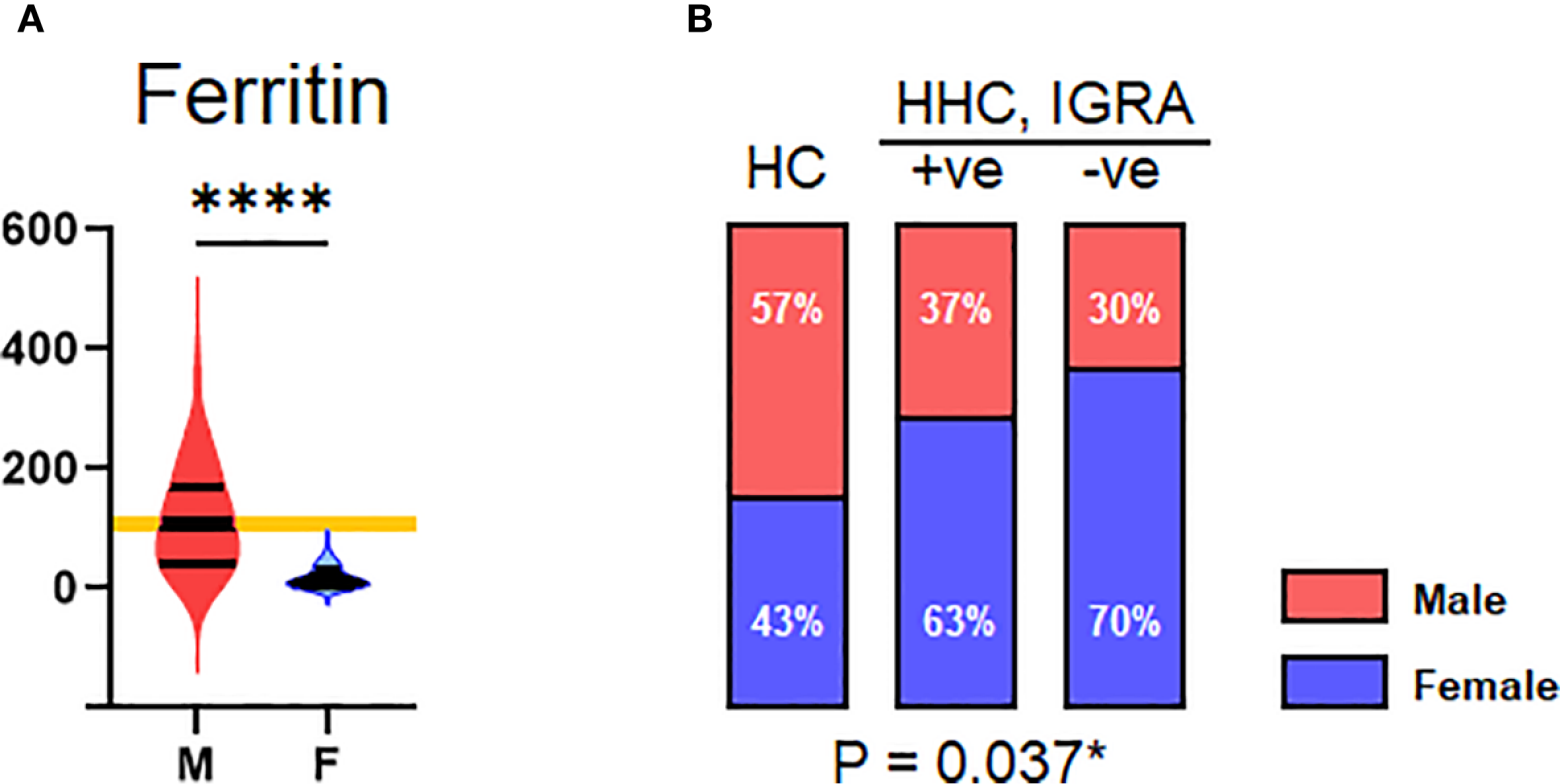
Gender. **(A)** Distribution of serum ferritin across male and female genders. **(B)** Distribution of HC, HHC, IGRA+ve, and IGRA –ve by gender. Values indicated with *, and **** represent p<0.05, and <0.0001, respectively.

**Figure 6:**
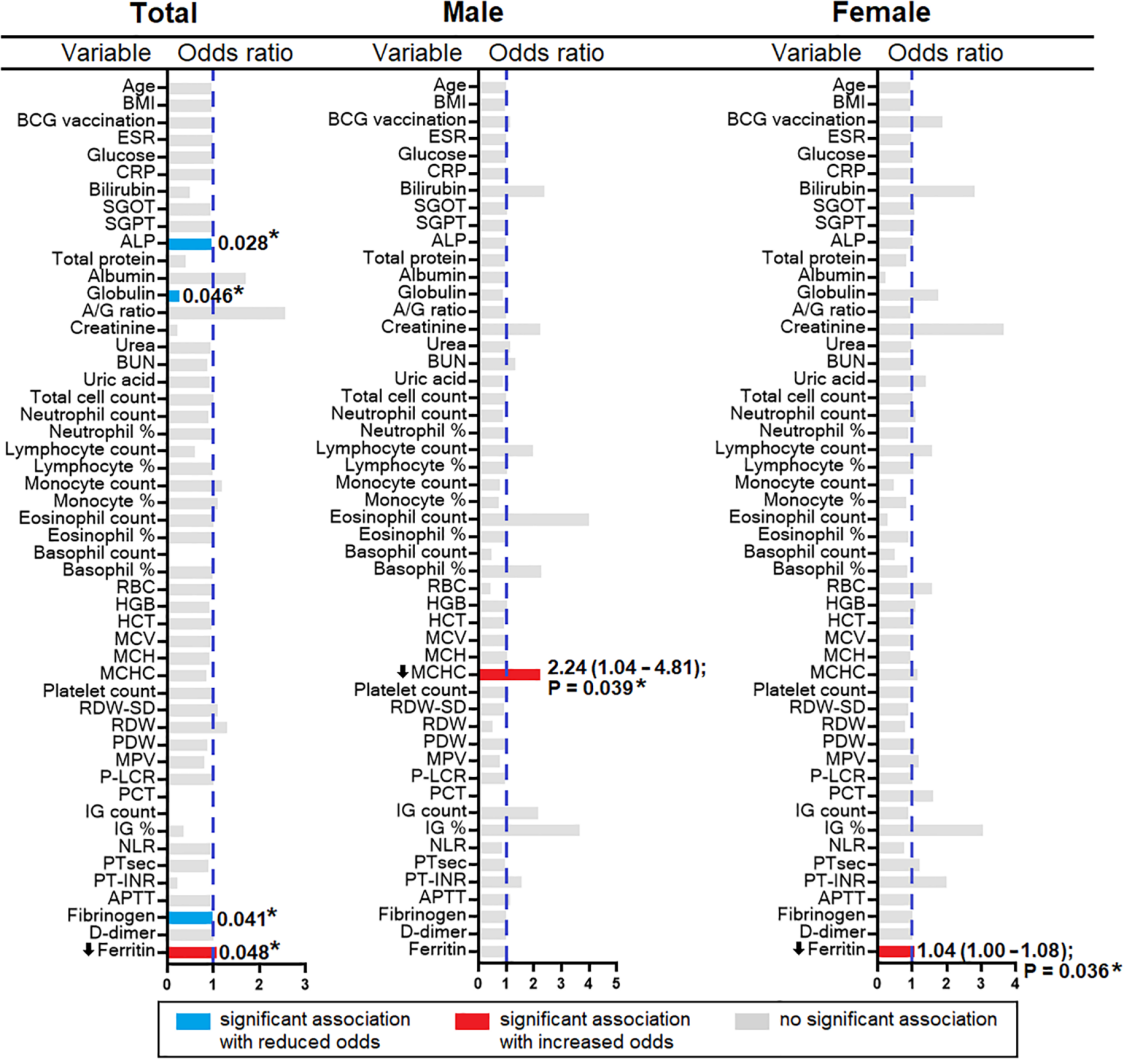
Factors associated with HHC/IGRA-ve stratified based on gender.

A decrease in ferritin level was observed in the HHC/IGRA-ve when compared to healthy controls and HHC/IGRA+ve (**Figure 2**). Ferritin level was found to be significantly lower in females when compared to males (**Figure 5**). A binary regression analysis was performed to determine the factors associated with HHC/IGRA-ve (**Figure 6**). The analysis showed that a decrease in ferritin was associated with increased odds of IGRA-ve in females by 1.04. As for males, MCHC was significantly associated, where every decrease of MCHC by 1 unit was associated with increased odds of IGRA-ve by 2.24. The detailed binary regression analysis of all the parameters associated with males and females is mentioned in **Supplementary Table 3**.

## Discussion

The global burden of LTBI was estimated to be 23% and the majority of them (80%) were from the WHO Southeast Asia, Western-Pacific, and African regions (20). There is an increasing need to identify novel approaches or host diagnostic biomarkers for improved diagnosis to prevent the risk of reactivation and subsequent transmission of the disease and to achieve the target of TB elimination by 2050. The biochemical and hematological indices serve as diagnostic and prognostic markers of several infectious diseases (21–23). These markers could aid in early clinical diagnosis and timely medical intervention. TB-infected patients undergo considerable immunohematological and biochemical changes during infection compared to healthy controls (24). Such parameters could potentially help in forecasting the onset of active TB and response to anti-TB treatment. However, the biological parameters of individuals with close contact with active TB patients who are either positive or negative for IGRA have not been investigated and largely remain ambiguous. To our knowledge, this is the first study to evaluate the biochemical and hematological parameters among HHCs of active TB patients. We previously reported the usefulness of plasma cytokines that could aid in diagnosing LTBI across HHCs/IGRA+ve (25). The study found an increased plasma CXCL8 and decreased MCP-1, TNF-α, and IFN-γ associated significantly with LTBI.

The study found ESR, D-dimer, PDW, MPV, and P-LCR to be significantly different from healthy controls and HHC/IGRA+ve individuals. The values obtained could potentially be used to establish reference ranges and to differentiate LTBI from healthy individuals, to aid clinical interpretation and decision-making.

The study found ESR levels among individuals with LTBI to be significantly higher in the HHC/IGRA+ve group. The diagnostic accuracy of ESR above the cut-off of 36.50 mm/hr, which indicates inflammation, although not reliable, is used to predict LTBI. High ESR (>40mm/hr) and thrombocytosis with platelet count (>500×10^3^/μL) have been associated with active TB infection; however, its use in LTBI has not been established (26). Despite low specificity due to increased levels observed across various conditions like cancer or traumatic ailments, ESR can provide valuable information on disease pathology involving inflammation and infection (27).

In our study, significantly higher PDW, MPV, and P-LCR values were seen in HHC/IGRA+ve individuals. The ROC analysis showed that among the three platelet indices, PDW and P-LCR showed a significant association with LTBI. Recent studies suggest that platelets play a vital role in innate defense against TB (28). An elevated platelet count, CRP, and ESR but not MPV were shown to be significantly associated with pulmonary TB cases (29). The elevated PDW, MPV, and P-LCR values indicate platelet activation and hypercoagulability. The clinical importance of infection-associated thrombosis has recently been recognized and is still not completely understood (30). A recent study showed the risk of myocardial infarction among people with LTBI. The study also showed a higher risk of developing hypertension among untreated LTBI+ve individuals compared to healthy controls (31). Studies have found that PDW and MPV were elevated in pulmonary TB (32, 33). Platelets were identified within the granulomas, and high platelet counts have been found to be associated with TB disease severity (34). Despite clear evidence on the association between TB and platelet indices reported previously (35), information is scarce on the characteristics of these indices in LTBI individuals and their role in reactivation. P-LCR is a marker that indicates the proportion of circulating platelets of more than 12 femtoliters. It identifies platelets that metabolically and enzymatically exhibit more significant activity than smaller ones. The present study indicates the association between P-LCR and LTBI.

Platelets are now increasingly recognized as active players in immune modulation and inflammation. Even during latency, there is a low-grade immune activation to MTB antigens that drives compensatory hematopoietic responses such as increased megakaryopoiesis in the bone marrow (36). This could lead to more large or immature platelets and therefore higher P-LCR. The pattern recognition receptors including Toll-like receptors and C-type lectin receptors, produced by platelets in response to interaction with MTB antigens, release pro-inflammatory cytokines that sustain granuloma stability (37). Prior studies have shown that P-LCR can serve as a diagnostic tool for hematologic disorders (38), such as chronic myeloid leukemia, and has been proven as a biomarker of myelodysplastic syndromes (39, 40). Most existing studies on platelet indices and TB have focused on active TB patients or general inflammatory conditions, not specifically IGRA-defined LTBI in household contacts. Our study showed a significant association with P-LCR, indicating subtler but still detectable platelet activation patterns, potentially manifesting as an increased proportion of large platelets (higher P-LCR) rather than overt thrombocytosis or MPV changes noted in active disease (41). D-dimer, a fibrin degradation product, serves as an indicator of thrombosis. Increased D-dimer indicates hypercoagulability in pulmonary TB (18). In this study, we showed D-dimer levels to be significantly higher in LTBI. However, this is contrary to other findings that have reported an average D-dimer level in LTBI patients diagnosed using the PPD test (42).

The present study showed that when combining ESR and P-LCR, the efficacy of diagnosing LTBI was high, with an AUC of 0.906. Additionally, Spearman’s correlation analysis revealed a significant positive correlation between ESR and other parameters such as CRP, ALT, and fibrinogen in LTBI cases. It could hence be construed that ESR and P-LCR as good surrogate diagnostic markers of subclinical inflammation associated with LTBI among HHCs. The ESR that is measured using the Westergren method and P-LCR through automated blood cell counting acts as a valuable surrogate diagnostic marker and, when adequately validated, could prove the effect on the diagnosis and subsequent clinical outcome of interest (43). Although these inflammatory markers cannot independently replace IGRA and TST, these tests could aid in LTBI, especially among high-risk individuals where rapid diagnosis is critical. Our study showed that the HHC/IGRA -ve individuals, albeit IGRA negative, biochemical profiles were similar to those in HHC/IGRA+ve individuals, except for serum ferritin and MCHC levels, indicating that they may have been in contact with TB cases or LTBI individuals.

The present study indicated the possible association of serum ferritin and MCHC levels with IGRA-ve. MCHC indicates the average concentration of hemoglobin in the RBCs. In this study, the binary regression revealed that MCHC (OR 2.240, 95% CI: 1.042 – 4.814, p=0.039) was found to be significantly associated with males in the IGRA-ve group. Ferritin levels were found to be significantly low (OR 1.037, 95% CI: 1.001 – 1.079, p=0.03) in the females who were IGRA-ve. The study conceivably had sex-imbalanced sampling, which could lead to unintentional gender bias as the study population was not an age- and sex-matched cohort, which is one of the limitations of the study.

Serum ferritin, a test of iron status, originates from damaged cells, reflecting cellular damage and therefore serves as an important inflammatory disease marker (44). Iron plays a crucial role as a cofactor in mycobacterial infection. The production of acute-phase proteins (APPs) in response to inflammation can stimulate or inhibit iron metabolism (45). Elevated serum ferritin (hyperferritinemia) is associated with different inflammatory diseases and their severity. A possible explanation lies in the differing iron metabolism profiles between infected and non-infected individuals. In LTBI (IGRA-positive), immune activation can induce hepcidin-mediated iron sequestration, leading to elevated ferritin as part of the anemia of inflammation. In contrast, IGRA-negative participants, particularly females, may reflect baseline physiological or nutritional iron status, with lower ferritin likely attributable to menstrual blood loss or dietary iron insufficiency rather than infection-related inflammation. Similarly, the association of lower MCHC in IGRA-negative males may relate to underlying hematological or nutritional variations rather than a direct protective effect against LTBI. A previous study showed decreased serum ferritin levels in active TB cases compared to the HHCs, suggesting the downregulation of NRAMP1 gene expression in TB cases (46). Other studies showed Hepcidin but not ferritin to be associated with TB disease severity and progression to active TB (47). However, a recent study among children infected with active and latent infection indicated elevated ferritin levels as a reliable marker for discriminating LTBI from active infection (48). This could possibly be due to the maintenance of iron balance that is critical for effective regulation in MTB, as excess iron can enhance survival, replication, and virulence (45, 49). The analysis was adjusted for sex as a covariate, and previous evidence indicates minimal sex-related variation in hematological and ferritin values within the studied population, suggesting the imbalance is unlikely to have influenced the results (50). Several confounding factors could influence the serum ferritin level, including diet, other causes of inflammation, underlying comorbidities, menstrual cycle, other hormonal changes, and environmental determinants. These factors were not considered in our analysis, and the association with the identified variation in the ferritin level is not known. Given the cross-sectional nature of our study, these findings should be interpreted cautiously and may reflect population-specific factors or residual confounding rather than causal relationships. We did not observe significant differences in hematological parameters like TC, neutrophils, lymphocytes, monocytes, basophils, eosinophils, platelet counts, or hemoglobin and hematocrit levels. This was in corroboration with other studies that found no association of blood cell counts with LTBI (51).

Our findings suggest that simple, routinely available hematological indices, such as ESR, PDW, MPV, and P-LCR, could serve as cost-effective adjunctive markers for identifying HHCs at higher risk of LTBI in resource-limited settings, potentially enabling earlier targeted screening and preventive interventions. While these markers cannot replace IGRA, their integration into clinical workflows could help prioritize individuals for confirmatory testing, thereby improving case detection efficiency.

The present study, however, suffered certain limitations. Firstly, the small sample size appears to limit the statistical power and generalizability of the findings to a broader population, which is particularly paramount given the diversity and variability within a larger population. Secondly, the lack of longitudinal follow-up restricts our ability to assess the long-term outcomes and deviations in the parameters studied over time. Thirdly, confounding factors that could influence the elevation of these inflammatory markers have not been studied. Fourthly, the cross-sectional design precludes establishing causal relationships, and the relatively small, single-center sample limits the generalizability of the findings to other populations. Furthermore, lack of external validation, unmatched controls and the assessed parameters that are closely linked to infection status at the time of sample collection present significant limitations. The findings are likely population-specific, and differences in genetic background, nutritional status, comorbidities, and TB exposure patterns across other demographic, geographic, or clinical settings may yield different associations. Therefore, this warrants future studies to validate the findings using larger and more diverse cohorts to ensure that the results are extrapolated across diverse demographics and clinical settings. Incorporating longitudinal follow-up is key to monitoring long-term outcomes and an improved understanding of the sustainability of the diagnostic biomarkers identified herein.

## Conclusions

The current study identified ESR and P-LCR as prominent surrogate diagnostic biomarkers of subclinical inflammation associated with LTBI. The hematological profile, especially ESR and P-LCR of IGRA-ve HHCs of active TB, was similar to that of LTBI, indicating that they might have been infected with MTB. The serum ferritin and MCHC were found to be associated with IGRA-ve. Additional investigations are warranted to arrive at more accurate and definitive explanations of the significant findings observed herein.

## Data Availability

The original contributions presented in the study are included in the article/**Supplementary Material**. Further inquiries can be directed to the corresponding authors.

## References

[1] Kumar V Nazli Khatib M Verma A Lakhanpal S Ballal S Kumar S. Tuberculosis in South Asia: A regional analysis of burden, progress, and future projections using the global burden of disease (1990-2021). J Clin Tuberc Other Mycobact Dis. (2024) 37:100480. doi: 10.1016/J.JCTUBE.2024.100480, PMID: 39507205 PMC11539151

[2] Selvavinayagam ST Sankar G Yong YK Sankar S Zhang Y Tan HY. Association of clinical laboratory parameters with latent tuberculosis infection among healthcare workers of primary health centers-A cross-sectional observational study. PLOS Glob Public Health. (2025) 5:e0004873. doi: 10.1371/journal.pgph.0004873, PMID: 40577412 PMC12204540

[3] Falzon D Zignol M Bastard M Floyd K Kasaeva T. The impact of the COVID-19 pandemic on the global tuberculosis epidemic. Front Immunol. (2023) 14:1234785/BIBTEX. doi: 10.3389/FIMMU.2023.1234785/BIBTEX, PMID: 37795102 PMC10546619

[4] Mack U Migliori GB Sester M Rieder HL Ehlers S Goletti D. LTBI: latent tuberculosis infection or lasting immune responses to M. tuberculosis? A TBNET consensus statement. Eur Respir J. (2009) 33:956–73. doi: 10.1183/09031936.00120908, PMID: 19407047

[5] Miggiano R Rizzi M Ferraris DM. Mycobacterium tuberculosis pathogenesis, infection prevention and treatment. Pathogens. (2020) 9:385. doi: 10.3390/PATHOGENS9050385, PMID: 32443469 PMC7281116

[6] Sharma SK Vashishtha R Chauhan LS Sreenivas V Seth D. Comparison of TST and IGRA in diagnosis of latent tuberculosis infection in a high TB-burden setting. PloS One. (2017) 12:e0169539. doi: 10.1371/JOURNAL.PONE.0169539, PMID: 28060926 PMC5218498

[7] Herrera V Perry S Parsonnet J Banaei N. Clinical application and limitations of interferon-gamma release assays for the diagnosis of latent tuberculosis infection. Clin Infect Dis. (2011) 52:1031–7. doi: 10.1093/CID/CIR068, PMID: 21460320

[8] Leo S Narasimhan M Rathinam S Banerjee A. Biomarkers in diagnosing and therapeutic monitoring of tuberculosis: a review. Ann Med. (2024) 56:2386030. doi: 10.1080/07853890.2024.2386030, PMID: 39097795 PMC11299445

[9] Wang SQ Inci F De Libero G Singhal A Demirci U. Point-of-care assays for tuberculosis: role of nanotechnology/microfluidics. Biotechnol Adv. (2013) 31:438–49. doi: 10.1016/J.BIOTECHADV.2013.01.006, PMID: 23357365 PMC3602163

[10] Barter DM Agboola SO Murray MB Bärnighausen T. Tuberculosis and poverty: the contribution of patient costs in sub-Saharan Africa–a systematic review. BMC Public Health. (2012) 12:980. doi: 10.1186/1471-2458-12-980, PMID: 23150901 PMC3570447

[11] Mariah Benedict Raj P Ganta GK Durai Singh C Muthusamy R. Efficacy of various tuberculosis treatment regimens at a tertiary health care center in south India: A retrospective study. Cureus. (2024) 16:e64496. doi: 10.7759/CUREUS.64496, PMID: 39139313 PMC11320887

[12] Singh S Zahiruddin QS Lakhanpal S Ballal S Kumar S Bhat M. Wealth-based inequalities in tuberculosis prevalence among households having children and young adults in India: insights from Indian demographic and health surveys (2015–2021). BMC Infect Dis. (2025) 25:21. doi: 10.1186/s12879-024-10301-7, PMID: 39755594 PMC11700442

[13] Rohini K Surekha Bhat M Srikumar PS Mahesh Kumar A. Assessment of hematological parameters in pulmonary tuberculosis patients. Indian J Clin Biochem. (2016) 31:332–5. doi: 10.1007/S12291-015-0535-8, PMID: 27382206 PMC4910852

[14] Leboueny M Maloupazoa Siawaya AC Bouanga LDJ Mvoundza Ndjindji O Mveang Nzoghe A Djoba Siawaya JF. Changes of C-reactive protein and Procalcitonin after four weeks of treatment in patients with pulmonary TB. J Clin Tuberc Other Mycobact Dis. (2023) 31:100348. doi: 10.1016/J.JCTUBE.2023.100348, PMID: 36714271 PMC9879784

[15] Stefanescu S Cocoş R Turcu-Stiolica A Shelby ES Matei M Subtirelu MS. Prediction of treatment outcome with inflammatory biomarkers after 2 months of therapy in pulmonary tuberculosis patients: Preliminary results. Pathogens. (2021) 10:789. doi: 10.3390/PATHOGENS10070789/S1, PMID: 34206598 PMC8308673

[16] Robson SC White NW Aronson I Woollgar R Goodman H Jacobs P. Acute-phase response and the hypercoagulable state in pulmonary tuberculosis. Br J Haematol. (1996) 93:943–9. doi: 10.1046/J.1365-2141.1996.D01-1722.X, PMID: 8703831

[17] Min W Zi-Feng J Jian-Lin X Hao-Hui F. Role of the fibrinogen degradation products and D-dimer in the differential diagnosis of pulmonary tuberculosis and community-acquired pneumonia. Clin Lab. (2018) 64:135–40. doi: 10.7754/CLIN.LAB.2017.170720, PMID: 29479876

[18] Kager LM Blok DC Lede IO Rahman W Afroz R Bresser P. Pulmonary tuberculosis induces a systemic hypercoagulable state. J Infect. (2015) 70:324–34. doi: 10.1016/J.JINF.2014.10.006, PMID: 25455017

[19] Mitroi DM Balteanu MA Cioboata R Vlasceanu SG Zlatian OM Catana OM. Hypercoagulability in tuberculosis: pathophysiological mechanisms, associated risks, and advances in management—A narrative review. J Clin Med. (2025) 14:762. doi: 10.3390/JCM14030762, PMID: 39941433 PMC11818899

[20] Houben RMGJ Dodd PJ. The global burden of latent tuberculosis infection: A re-estimation using mathematical modelling. PloS Med. (2016) 13:e1002152. doi: 10.1371/JOURNAL.PMED.1002152, PMID: 27780211 PMC5079585

[21] Alizad G Ayatollahi AA Shariati Samani A Samadizadeh S Aghcheli B Rajabi A. Hematological and biochemical laboratory parameters in COVID-19 patients: A retrospective modeling study of severity and mortality predictors. BioMed Res Int. (2023) 2023:7753631. doi: 10.1155/2023/7753631, PMID: 38027038 PMC10676280

[22] Ezhilarasan D. Antitubercular drugs induced liver injury: an updated insight into molecular mechanisms. Drug Metab Rev. (2023) 55:239–53. doi: 10.1080/03602532.2023.2215478, PMID: 37218081

[23] Gowhari Shabgah A Abdelbasset WK Sulaiman Rahman H Bokov DO Suksatan W Thangavelu L. A comprehensive review of IL-26 to pave a new way for a profound understanding of the pathobiology of cancer, inflammatory diseases and infections. Immunology. (2022) 165:44–60. doi: 10.1111/imm.13424, PMID: 34716913

[24] Gebreweld A Fiseha T Kebede E Tamir Z Gebremariam B Miruts F. Immuno-hematological and biochemical changes in patients with tuberculosis in dessie comprehensive specialized hospital, dessie, Ethiopia. J Blood Med. (2024) 15:147–55. doi: 10.2147/JBM.S445857, PMID: 38532889 PMC10964777

[25] Selvavinayagam ST Aswathy B Yong YK Frederick A Murali L Kalaivani V. Plasma CXCL8 and MCP-1 as surrogate plasma biomarkers of latent tuberculosis infection among household contacts-A cross-sectional study. PloS Global Public Health. (2023) 3:e0002327. doi: 10.1371/JOURNAL.PGPH.0002327, PMID: 37992019 PMC10664947

[26] Shah AR Desai KN Maru AM. Evaluation of hematological parameters in pulmonary tuberculosis patients. J Family Med Prim Care. (2022) 11:4424. doi: 10.4103/JFMPC.JFMPC_2451_21, PMID: 36353004 PMC9638606

[27] Litao MKS Kamat D. Erythrocyte sedimentation rate and C-reactive protein: how best to use them in clinical practice. Pediatr Ann. (2014) 43:417–20. doi: 10.3928/00904481-20140924-10, PMID: 25290132

[28] Carranza C Pedraza-Sanchez S de Oyarzabal-Mendez E Torres M. Diagnosis for latent tuberculosis infection: new alternatives. Front Immunol. (2020) 11:2006. doi: 10.3389/FIMMU.2020.02006, PMID: 33013856 PMC7511583

[29] Gunluoglu G Yazar EE Veske NS Seyhan EC Altin S. Mean platelet volume as an inflammation marker in active pulmonary tuberculosis. Multidiscip Respir Med. (2014) 9:11. doi: 10.1186/2049-6958-9-11, PMID: 24581084 PMC3995664

[30] Beristain-Covarrubias N Perez-Toledo M Thomas MR Henderson IR Watson SP Cunningham AF. Understanding infection-induced thrombosis: lessons learned from animal models. Front Immunol. (2019) 10:2569/PDF. doi: 10.3389/FIMMU.2019.02569/PDF, PMID: 31749809 PMC6848062

[31] Mandieka E Saleh D Chokshi AK Rivera AS Feinstein MJ. Latent tuberculosis infection and elevated incidence of hypertension. J Am Heart Assoc. (2020) 9:19144. doi: 10.1161/JAHA.120.019144/ASSET/897ABC50-9235-4633-B0F6-8AE1095BCC51/ASSETS/GRAPHIC/JAH35760-FIG-0001.PNG, PMID: 33263262 PMC7955375

[32] Tozkoparan E Deniz O Ucar E Bilgic H Ekiz K. Changes in platelet count and indices in pulmonary tuberculosis. Clin Chem Lab Med. (2007) 45:1009–13. doi: 10.1515/CCLM.2007.194, PMID: 17867990

[33] Shankaralingappa A Tummidi S Arun Babu T. Diagnostic value of platelet indices in COVID 19 infection: a case-control study from a single tertiary care center. Egypt J Intern Med. (2022) 34:35. doi: 10.1186/S43162-022-00123-X, PMID: 35382491 PMC8972661

[34] Nancy Hilda J Venkataraman A Thiruvengadam K Brindha B Karthick M Subha S. Evaluation of platelet indices as markers of tuberculosis among children in India. ERJ Open Res. (2024) 10:00734–2023. doi: 10.1183/23120541.00734-2023, PMID: 38410718 PMC10895425

[35] Kirwan DE Chong DLW Friedland JS. Platelet activation and the immune response to tuberculosis. Front Immunol. (2021) 12:631696. doi: 10.3389/FIMMU.2021.631696, PMID: 34093524 PMC8170316

[36] Wang L Kuang Y Zeng Y Wan Z Yang S Li R. Association of systemic inflammation response index with latent tuberculosis infection and all-cause mortality: a cohort study from NHANES 2011-2012. Front Immunol. (2025) 16:1538132. doi: 10.3389/FIMMU.2025.1538132, PMID: 40046059 PMC11880221

[37] Cognasse F Nguyen KA Damien P McNicol A Pozzetto B Hamzeh-Cognasse H. The inflammatory role of platelets via their TLRs and siglec receptors. Front Immunol. (2015) 6:83. doi: 10.3389/FIMMU.2015.00083, PMID: 25784910 PMC4345914

[38] Turk U Tengiz I Ozpelit E Celebiler A Pekel N Ozyurtlu F. The relationship between platelet indices and clinical features of coronary artery disease. Kardiol Pol. (2013) 71:1129–34. doi: 10.5603/KP.2013.0293, PMID: 24297710

[39] Chen Q Chen Y Zhang Y Zhang L Chen K He Z. Prognostic impact of platelet-large cell ratio in myelodysplastic syndromes. Front Oncol. (2022) 12:846044. doi: 10.3389/FONC.2022.846044, PMID: 35433406 PMC9010610

[40] Kabutomori O Kanakura Y Iwatani Y. Increase in platelet-large cell ratio in chronic myeloid leukemia. Leuk Res. (2001) 25:873. doi: 10.1016/S0145-2126(01)00017-0, PMID: 11532520

[41] Ştefanescu S Cocoş R Turcu-Stiolica A Mahler B Meca AD Giura AMC. Evaluation of prognostic significance of hematological profiles after the intensive phase treatment in pulmonary tuberculosis patients from Romania. PloS One. (2021) 16:e0249301. doi: 10.1371/JOURNAL.PONE.0249301, PMID: 33793598 PMC8016233

[42] Shitrit D Izbicki G Bar-Gil Shitrit A Raz M Sulkes J Kramer MR. Normal D-dimer levels in patients with latent tuberculosis infection. Blood Coagul Fibrinolysis. (2005) 16:85–7. doi: 10.1097/00001721-200501000-00014, PMID: 15650552

[43] Katz R. Biomarkers and surrogate markers: an FDA perspective. NeuroRx. (2004) 1:189. doi: 10.1602/NEURORX.1.2.189, PMID: 15717019 PMC534924

[44] Kell DB Pretorius E. Serum ferritin is an important inflammatory disease marker, as it is mainly a leakage product from damaged cells. Metallomics. (2014) 6:748–73. doi: 10.1039/C3MT00347G, PMID: 24549403

[45] Moreira AC Mesquita G Gomes MS. Ferritin: an inflammatory player keeping iron at the core of pathogen-host interactions. Microorganisms. (2020) 8:589. doi: 10.3390/MICROORGANISMS8040589, PMID: 32325688 PMC7232436

[46] Wu L Deng H Zheng Y Mansjö M Zheng X Hu Y. An association study of NRAMP1, VDR, MBL and their interaction with the susceptibility to tuberculosis in a Chinese population. Int J Infect Dis. (2015) 38:129–35. doi: 10.1016/J.IJID.2015.08.003, PMID: 26261060

[47] Hella J Cercamondi CI Mhimbira F Sasamalo M Stoffel N Zwahlen M. Anemia in tuberculosis cases and household controls from Tanzania: Contribution of disease, coinfections, and the role of hepcidin. PloS One. (2018) 13:e0195985. doi: 10.1371/JOURNAL.PONE.0195985, PMID: 29677205 PMC5909902

[48] Comella-Del-Barrio P Abellana R Villar-Hernández R Coute MDJ Mingels BS Aliaga LC. A model based on the combination of IFN-γ, IP-10, ferritin and 25-hydroxyvitamin D for discriminating latent from active tuberculosis in children. Front Microbiol. (2019) 10:1855. doi: 10.3389/FMICB.2019.01855, PMID: 31474956 PMC6702835

[49] Pandey R Rodriguez GM. A ferritin mutant of mycobacterium tuberculosis is highly susceptible to killing by antibiotics and is unable to establish a chronic infection in mice. Infect Immun. (2012) 80:3650. doi: 10.1128/IAI.00229-12, PMID: 22802345 PMC3457556

[50] Rushton DH Barth JH. What is the evidence for gender differences in ferritin and haemoglobin? Crit Rev Oncol Hematol. (2010) 73:1–9. doi: 10.1016/J.CRITREVONC.2009.03.010, PMID: 19394859

[51] Takenami I Loureiro C MaChado A Emodi K Riley LW Arruda S. Blood cells and interferon-gamma levels correlation in latent tuberculosis infection. ISRN Pulmonol. (2013) 2013:1–8. doi: 10.1155/2013/256148, PMID: 24040564 PMC3769793

